# Integration of leadership training for graduate and medical students engaged in translational biomedical research: Examining self-efficacy and self-insight

**DOI:** 10.1017/cts.2018.9

**Published:** 2018-06-06

**Authors:** Celia Chao, Kevin Wooten, Heidi Spratt, Huda Sarraj, Judith Aronson, Jonathan Hommel, Ross Ungerleider, Jamie D. Ungerleider, Mark R. Hellmich

**Affiliations:** 1 Department of Surgery, University of Texas Medical Branch, Galveston, TX, USA; 2 Human Pathophysiology and Translational Medicine Graduate Program, University of Texas Medical Branch, Galveston, TX, USA; 3 Department of Sociology, Institute for Translational Sciences, University of Texas Medical Branch, Galveston, TX, USA; 4 Department of Administrative Sciences, School of Business, University of Houston, Clear Lake, TX, USA; 5 Preventive Medicine and Community Health, University of Texas Medical Branch, Galveston, TX, USA; 6 Department of Pathology, University of Texas Medical Branch, Galveston, TX, USA; 7 Department of Pharmacology and Toxicology, University of Texas Medical Branch, Galveston, TX, USA; 8 Pediatric Cardiac Surgery, Driscoll Children’s Hospital, Corpus Christi, TX, USA; 9 Co-Director, System’s Evolution Relationship Coaching for Professionals, Corpus Christi, TX, USA

**Keywords:** Leadership, team science, graduate students, medical students, interprofessional collaboration

## Abstract

**Introduction:**

Formal training in team leadership is not taught in biomedical research graduate training programs or medical schools.

**Methods:**

We piloted a Leadership Training Workshop for graduate biomedical and medical students enrolled in our Interprofessional Research Design Course.

**Results:**

The Kane–Baltes self-efficacy survey demonstrated improved leadership skills (median scores pretraining and post-training were 71 and 76.6; paired *t*-test, *p*=0.04).

**Conclusions:**

Most students demonstrated significant improvement in self-awareness pertaining to their own innate leadership styles.

## Introduction

The successful biomedical researcher will need to lead research teams effectively in order to understand relevant gaps in knowledge and solve complex problems required to advance the broad field of medicine. Multidisciplinary and interprofessional team science is increasingly recognized as a key driver for the most impactful and efficient way to translate scientific knowledge to improve public health [1]. While it is widely believed that team and leadership training can improve interdisciplinary team science [2], it is generally acknowledged that currently there is insufficient training and curriculum in the science of teamwork and leadership [3].

Formal training in team leadership and project management is not routinely taught in biomedical research graduate training programs or medical schools [4]. By default, students often model their leadership style and behaviors based on that of their mentors, consistent with the traditional apprenticeship model of education. This method of learning may be neither adequate nor effective. As research teams are becoming more interdisciplinary, codifying the training of graduate students and/or medical students in professional skills related to leadership styles, project management, and conflict resolution in interdisciplinary research teams are important training goals [5].

We report our experience piloting a leadership training program at the University of Texas Medical Branch (UTMB) during the Summer Term of 2017 with graduate students enrolled in the Human Pathophysiology and Translational Medicine Graduate Program’s core course, Translational Research Design and Interprofessional Skills Development (shortened, Interprofessional Research Design), and first-year medical students in the Translational Research Track. The structured curricula we developed and implemented focused more on processes rather than outcomes, given the time limitation of the course and our instructional objectives. We devoted 12 hours of a 7-week course toward leadership training. Following best practices from team science educators [6], we adapted exercises from leadership workshops for healthcare providers, which have been shown to be effective and desirable, as measured by outcomes such as patient mortality and prevalence of medical errors [7]. Our goal was to integrate the fundamentals of leadership into a novel interdisciplinary research design course providing not only the appropriate context for an authentic learning experience as a multidisciplinary research team member, but also to enhance each student’s ability to lead a translational science team.

Since 2012, the Interprofessional Research Design Course partnered predoctoral Ph.D. students with medical students who also participated in summer research rotations. The overall course goals are to promote: (1) development of interprofessional collaborative skills, (2) skills of research project development and study design, (3) oral and written presentation skills, and beginning in 2017, (4) understanding leadership styles that foster collaboration in translational research projects. The course uses a combination of guided inquiry, team-based learning, workshop and seminar formats. These varied active learning styles lead students through the processes related to the conduct and performance relevant to team science: formation and refinement of a study hypothesis or research question, selection of (an) appropriate experimental design(s), and development of data evaluation plan(s).

The final product of the course is a multidisciplinary, collaborative research project proposal on a translational topic in the NIH R21 format. Each group of students (1 Ph.D. and 2–3 M.D. students per group) collectively was required to create a translational research proposal. Each student negotiated their individual contributions toward the final product in a team contract, and documented (assessed) the meaningful contributions of each member, including their own, in the final product at the end of the course. Their research proposal was graded based on scientific soundness, and evidence of interdisciplinary, interprofessional collaborative interactions (includes consultation with a biostatistician).

## Methods

### Study Design

The Institutional Review Board at UTMB reviewed and approved this project. We designed a 12-hour leadership training and used pre–post mixed methods design to assess the effects of the intervention.

### Sample

A total of 17 students (9 females and 8 males) were enrolled in the Interprofessional Research Design Course (12 Medical students, 4 Ph.D. students, 1 M.D.-Ph.D. student) during the Summer Term (May–June) of 2017 at the UTMB Graduate School of Basic Sciences and the School of Medicine.

### Procedures

Students took a pretest before attending class and then attended a 12-hour session on leadership training over 1.5 days (intervention). At the beginning of the training, every participant took the Myers–Briggs personality inventory [8], obtained a Blake–Mouton grid score [9], and completed the Thomas–Kilmann Conflict Style Index [10]. These 3 tests were used as tools to help the students develop awareness of their leadership styles. In the workshop, students worked in groups of similar personality types to explain their leadership styles to members of the other different personality types. This exercise provided the opportunity for everyone to understand their own personality type as well as that of their classmates. In-class exercises were designed to be experiential, rather than didactic, and focused on appreciating that leadership is one’s self-development in service of others. Other exercises explored ways to better understand the motivations and perspectives of their colleagues, as well as ways to communicate effectively in a team. They were introduced to ways to explore those differences in order to better dialog with their team members, and incorporate ideas into a construct that best represents the “collective wisdom” of the team. At the end of the 7-week interprofessional research design course, the students took the test again (post-test).

Students were also required to have team contract agreements, which delineated each member’s proposed contributions and timeline to completion of tasks before engaging in the group exercises. At the end of the class, the students were told to document the meaningful contributions of each team member, relative to their own contributions.

### Measures

Research based on self-efficacy theory shows that efficacious persons are motivated, resilient, and goal oriented [11], and this has been extended to general leadership [12], leader effectiveness [13], and group functioning [14]. The pretest and post-test embedded the Kane–Baltes Leadership Self Efficacy Survey [15], which has been developed specifically to measure self-perceived capability to function as a team leader. Initial development [15] of this survey reported a high reliability (α=0.93), and subsequent uses [16, 17] equally high (α=0.90, α=0.95). We found the reliability of this survey in this study to be similar (α=0.88). The survey has been used as an experimental validity check [15], and has been shown to be predictive of team leadership [17], as well as goal and strategy setting in team settings [16].

The pre-post-leadership workshop test asked open-ended questions, probed whether the students recognized their own leadership style, and whether they are aware of communication methods that minimize conflict and maximize team productivity. Although the Myers-Briggs, the Blake–Mouton, and the Thomas–Kilmann instruments were used as learning tools to help students gain awareness of their own innate leadership styles, we also used these 3 tests as measures (preworkshop and postworkshop) of whether the students were self-aware of their authentic leadership style.

Lastly, the students were required to submit a final writing assignment reflecting on a meaningful event in the team process that will carry influence with them beyond the Interprofessional Research Design course. From the essays, we identified 4 major themes.

### Statistics

The pretest and post-test answers were evaluated using the paired samples *t*-test (SPSS Version 22.0., Armonk, NY, USA). Since this is a practical intervention, we are interested in detecting large effect sizes for a difference between preassessment and postassessment values. Our power analysis indicates that we have sufficient (at least 80%) power to detect significant differences for Questions 4 and 5 which have large effect sizes (Table 1). For the rest of our questions, we likely do not have the power needed to detect a difference due to smaller effect sizes (0.5 and smaller). Cronbach’s α was calculated for the reliability of the post-test questions using the alpha function with the R psych package [18].

**Table 1:** Preclass and postclass Kanes–Baltes self-efficacy test results

Question	Mean±SD	Median	Paired *t*-test *p*-value
1. I have the necessary skills to perform well as a leader across different group settings	Pre 64.35±16.97	68	0.82
	Post 71.29±12.98	69	
2. I can motivate group members to accomplish a biomedical research project	Pre 66.18±14.93	67	0.489
	Post 68.71±19.97	77	
3. I have the skills to build group members’ confidence	Pre 69.35±11.01	71	0.556
	Post 71.82±20.02	71	
4. I have the skills to develop teamwork toward accomplishing a group biomedical research project	Pre 63.82±17.06	61	0.005*
	Post 78.53±12.58	85	
5. I can “take charge” when necessary in steering a team project	Pre 68.47±19.53	78	0.010*
	Post 82.06±16.34	83	
6. I can communicate effectively with team members	Pre 74.88±10.83	75	0.83
	Post 74.00±17.45	72	
7. I can develop effective task strategies to help accomplish the team goals	Pre 72.65±10.25	71	0.358
	Post 76.41±13.24	75	
8. I have the skills to assess the strengths and weaknesses of the group	Pre 75.94±19.16	83	0.611
	Post 78.41±14.96	80	
9. All 8 questions above	Pre 69.46±10.34	71	0.044*
	Post 75.15±11.94	76.6	

*Statistical significance <0.05.

## Results

Table 1 shows that among the 8 self-efficacy questions administered, 2 questions showed statistically significant higher scoring after leadership training. These 2 questions are more temporally relevant and focus on the awareness that participants acquired from the 12-hour class: “I have the skills to develop…” and “I can take charge….”

In a series of open-ended optional questions, students were asked to comment on leadership questions listed in Table 2. In general, the preworkshop answers were more superficial and generalized, whereas the postworkshop answers were more specific and insightful. For example, Participant 4 did not provide a written response to the quoted comment on leadership in Q 2; however, his postworkshop statement embodied the ideal answer.

**Table 2:** Examples of open-ended questions about leadership; preleadership and postleadership workshop results

Open-ended qualitative questions	Preworkshop answer	Postworkshop answer
Q 1: How do personality type relate to leadership ability?	Participant 1: “The personality type and leadership style of an individual shows how they will lead a group or act in a team setting. Both of these things show how people present themselves to a group”	Participant 1: “I think your leadership style when you’re stressed is as important as your leadership style when everything is going well. Your leadership styles at your worst are important to learn and cope with”
	Participant 2: “I actively try to be open-minded and willing to co-operate because those characteristics seem to facilitate the exploration of ideas…”	Participant 2: “I have discovered I am much less patient than I initially thought I was. When team members contribute, I appreciate it tremendously, and I enjoy incorporating their ideas. However, I resort to a commanding style when people consistently fail to meet expectations”
Q 2: Comment on the statement: “Structure and Process create outcomes. Every system is perfectly designed to give you the results you get and leaders have to be leaders of process (“drivers”) in order to achieve desired outcomes”	Participant 3: “Every research should have a plan and structure understandable and agreeable by its members so they know what are expected of them and the PI (principal investigator) should make sure that the plan is followed”	Participant 3: “No system is perfect to derive the desired outcome. Team work is a dynamic process and needs constant monitoring and changing if needed”
	Participant 4: No preworkshop comment	Participant 4: “I think leaders should be more people-oriented than process-oriented. People can take care of processes as part of their work, but relationships between people are complex and managing them might be appropriate for a leader”

Preworkshop, most students could not characterize their personal leadership style. After the students were exposed to Myers–Briggs, the Blake–Mouton, and the Thomas–Kilmann instruments, they learned to recognize their own leadership style preferences and appreciate the differing styles of their team members. As expected, in the pretest, many students did not know their own Myers–Briggs personality type; 53% of students did not know which personality qualities presented the greatest challenges that could lead to conflict. The majority of students were also unaware of their preferred conflict resolution style until they were introduced to the Blake–Mouton grid score and the Thomas–Kilmann Conflict Style Index. In the post-test, all participants knew their personality type, their personality under stress and their preferred conflict resolution style. As a result of exposure to the 3 tools, they began to recognize and think actively about managing their own predispositions, particularly when they obstruct their own ability to lead others.

In the final writing assignment, students reflected on a meaningful event in the team process that will carry influence with them beyond the Interprofessional Research Design course. In reflecting on their entire learning experience, 4 major themes predominated in their essays. They learned/experienced: (1) the development of interprofessional respect and learning from their peers (9/17 students; 53%), (2) learning to trust their teammates (7/17, 42%), (3) good communication and use of a team contract (12/17, 71%), and (4) personal growth and development (9/17, 53%). Overall, participants understand that a leader in team science is one who creates an environment for intellectual exchange of information, encourages risk-taking and problem solving, and develops strategies for overcoming challenges associated with merging scholars from diverse backgrounds. Further, these qualities are attainable after establishing trust, respect, and a mutual set of ground-rules by which all team members follow.

## Discussion

Over the past years, students who have participated in the Interprofessional Design course have provided overall positive feedback being engaged in a team science project. However, before 2017, self-reflection essays by some prior students revealed that not all team members carried their weight. Working in a team effectively requires a different skill set compared with working alone on an assignment, leading to predictable discord among team members. In 2017, we implemented a leadership workshop so that all students can be introduced to skills that will allow them to work within the context of a scientific team [2]. At times, a specific team member took the lead on one aspect of the project, as stated in their team contracts. Students learned to appreciate collaboration from other members of the team and integrated their unique perspectives for the shared common goals.

Postworkshop analysis revealed that students’ demonstrated improvement in leadership self-efficacy, developed an awareness of their own individual authentic leadership styles as well as understanding that of their colleagues’ styles, and further recognized that their styles differed under nonstressed versus stressed situations. The results of this study extend previous studies that involved developing leadership capacities in healthcare organizations [19], and also confirm prior findings that self-efficacy can be influenced by training [20]. The 12-hour introduction to leadership addresses the lack of team leadership courses [4], as well as a call by the National Research Council [21] to extend areas such as leadership theory into team science training. Future research related to developing interprofessional and leadership skills should explore more fully the effects of self-efficacy upon broader skill acquisition and retention.

Given that the current course design significantly influenced student beliefs concerning teamwork skills required to be successful and beliefs concerning taking charge of a project, additional research should explore other training designs that might influence other components of leadership self-efficacy (e.g., skills to perform well as a leader across different group settings). Perhaps the key to improving self-efficacy scores that seem less temporally related to the training design might involve implementation of a longitudinal leadership-coaching model.
